# POSTOPERATIVE DELIRIUM: A SURVEY OF PERCEPTIONS AND KNOWLEDGE AMONG SURGICAL PROVIDERS

**DOI:** 10.1093/geroni/igae098.1459

**Published:** 2024-12-31

**Authors:** Julia Berian, Ben Zarzaur, Sanjay Mohanty, Farah Kaiksow, Blair Golden

**Affiliations:** University of Wisconsin Madison, Madison, Wisconsin, United States; University of Wisconsin Madison, Madison, Wisconsin, United States; Indiana University School of Medicine, Indianapolis, Indiana, United States; University of Wisconsin School of Medicine and Public Health, Madison, Wisconsin, United States; University of Wisconsin Madison School of Medicine and Public Health, Madison, Wisconsin, United States

## Abstract

Postoperative delirium is associated with delayed recovery and cognitive decline. Despite increasing awareness among medical providers, understanding of delirium among surgical providers remains unknown. We aimed to evaluate knowledge and confidence in identifying and managing postoperative delirium, and to determine surgeons’ perceptions of the relative importance of postoperative delirium. A 14-question survey was distributed to surgeons and surgical advanced practice providers (APP) after validation through cognitive interviews. Eighty-five of 267 responded (31%), more attending surgeons (67%) than APPs (33%). Most had >5 years of experience (73%). Respondents estimated postoperative delirium rates from 0% to 65%, with the average varying by field from 5% (Endocrine surgery) to 33% (Trauma and Critical Care). Most had received formal delirium training (66%) and felt confident in identifying (80%) and managing (65%) delirium. Postoperative delirium was identified by nursing concerns (89%) or clinical gestalt (82%), with few reporting the use of validated tools (23%). Out of 6 common postoperative complications, surgical providers ranked postoperative delirium 3rd (28%) or 4th (25%), below cardiac complications (78%) and above superficial site infections (53%). The most concerning consequence of delirium was mortality (62%), with few concerned about psychological distress (16%). Most (60%) thought post-operative delirium had a limited effect on long-term health. Despite self-reported knowledge and high confidence in identifying and managing postoperative delirium, objective measures are rarely used, and long-term effects are thought to be minimal. Gaps in knowledge around delirium diagnosis and long-term health effects may contribute to the ongoing burden of delirium in older surgical patients.

